# Relationship between the stage of osteoarthritis before and six months after tibial tuberosity advancement procedure in dogs

**DOI:** 10.1371/journal.pone.0219849

**Published:** 2019-08-06

**Authors:** Stefania Pinna, Francesco Lanzi, Alessia Cordella, Alessia Diana

**Affiliations:** Department of Veterinary Medical Sciences, School of Agriculture and Veterinary Medicine, Alma Mater Studiorum, University of Bologna, Ozzano Emilia, Bologna, Italy; University of Camerino, ITALY

## Abstract

The present retrospective study evaluated the progression of osteoarthritis in stifle joints based on the radiographic grade of osteoarthritis (OA) scored in dogs with cranial cruciate ligament rupture. The aim of the study was to search for a correlation between the stage of radiographic osteoarthritis prior to surgery and the osteoarthritis progression occurring after the tibial tuberosity advancement (TTA) procedure. It was hypothesized that the procedure carried out in dogs in the early stages of OA could reduce the OA changes. A total of 190 X-ray images obtained from the medical records of 38 dogs were evaluated. The radiographic signs of osteoarthritis of 38 stifle joints were scored from 0 to 3 in 10 specific anatomic locations. The radiographs were divided into 4 groups based on the global scores: A) no-OA, B) mild-OA, C) moderate-OA, D) severe-OA; they were assessed prior to surgery, and 1, 2, 3 and 6 months post-operatively (T0, T1, T2, T3 and T6). There were no differences in osteoarthritis progression in Groups A and C at any time. Osteoarthritis changes from T0 to T6 were statistically significant in Group B. The OA changes in the anatomic locations were investigated. The most common anatomic sites for OA changes were the patella apex, the proximal and distal trochlear ridges, and the caudal aspect of the tibial plateau assessed before surgery. After surgery, the score increased in the first three locations in 10, 9 and 11 joints, respectively; instead, the progression of osteoarthritis in the caudal aspect of the tibial plateau occurred in 23 out of 38 stifle joints. The results indicated that the TTA procedure could be effective in slowing down the OA progression when carried out in the absence of or in the early stages of disease. Therefore, an early intervention may be suggested in clinical practice to obtain minimal or no progression 6 months postoperatively.

## Introduction

Cranial cruciate ligament (CCL) rupture is one of the most common causes of hind lameness and secondary osteoarthritis (OA) in the stifle joints of dogs due to the instability of the joint [1–3]. Many surgical procedures have been described in the treatment of CCL rupture; in spite of this, no surgical procedure seems to have achieved normal stifle biomechanics [4]. In recent years, various osteotomy techniques have been described. Currently tibial plateau levelling osteotomy (TPLO) and tibial tuberosity advancement (TTA) are the procedures most utilized for the treatment of CCL rupture [4,5]. They functionally stabilize the stifle joint by altering its geometry and biomechanics without mimicking the function of the CCL [6,7]. The TPLO and TTA procedures neutralize the cranial tibiofemoral shear force in two different ways. The first treatment reduces the tibial plateau angle by radial osteotomy of the proximal aspect of the tibia and rotation of the proximal fragment [8]. The second one advances the tibial tuberosity by means of an osteotomy of the tuberosity in the frontal plane with advancement of this bone fragment [9].

The progression of OA in CCL rupture has been assessed after conservative treatment, after extracapsular and intracapsular substitution techniques, and after osteotomy [10–12]. The evaluation of OA before and after a surgical procedure aims to assess the effectiveness of the treatment, but the radiographic signs of OA also vary with the chronicity of the disease [13,14]. Osteophytes have been observed microscopically at three days after experimental transection of the ligament, and radiographically at two to three weeks [14–16], although the development of osteophytes in confined experimental animals is likely to be slower than in natural settings in which the majority of osteophytes are formed as a result of joint instability in order to restore stability [13,16].

To the best of the authors’ knowledge, few studies have described the OA changes after TTA [17–19]. In these reports the OA was assessed using different scoring methods, but a single method is necessary to obtain comparable results from studies [20].

The aim of this retrospective study was to evaluate whether the stage of radiographic OA assessed prior to surgery could influence the OA progression occurring after the TTA procedure in dogs with spontaneous CCL rupture. It was hypothesized that TTA can lead to different results based on the degree of severity of the OA present before surgery. In particular, TTA carried out in dogs in the absence of or in the early stages of OA could slow the OA changes; early intervention would be ideal and suggested in clinical practice. Furthermore, the authors investigated the anatomic locations in the stifle joints in which the greatest radiographic signs of OA prior to surgery and their changes after TTA treatment were present.

## Materials and methods

The medical records of dogs with unilateral CCL rupture treated with a TTA procedure were retrospectively reviewed. Records and radiographs from October 2015 through November 2017 were obtained from the archives of the Department of Veterinary Medical Sciences, University of Bologna, Italy. The inclusion criteria were the presence of standard mediolateral projection radiographs [13,14] taken prior to surgery (T0) and at 1, 2, 3 and 6 months postoperatively (T1, T2, T3 and T6). At the time of the procedure, these radiographs had been taken in accordance with the standard operating protocol for the TTA procedure in the orthopedic unit in order to carry out preoperative planning [21] and evaluate the bone healing post-osteotomy. The inclusion criteria required a clinical follow up of 12 months, including body weight more than 15 kg, a diagnosis of complete CCL rupture based on physical examination in which the cranial drawer sign was evident in both the extension and the flexion of the stifle joint [22,23], no suspicion of meniscal tear detected by the tibial compression test carried out under axial loading through the full joint range of motion [24] and/or ultrasonography examination [25,26]. Clinical examination supported the hypothesis of complete rupture of the CCL and of intact menisci.

The medical records included the informed consent form signed by each dog owner as required for procedures involving anesthesia. Furthermore, the owners were informed regarding the surgical protocol which did not include the arthrotomy procedure, but that it would be performed only in recurrence of lameness or subsequent meniscal tear.

The exclusion criteria were incorrect/incomplete radiographic positioning, the presence of other concomitant orthopedic diseases, having undergone open arthrotomy and meniscal release, occurrence of major surgical complications, such as subsequent meniscal tear, and lameness in the contralateral hind limbs during the 12 months after surgery.

### Surgical procedures

All the surgical procedures were performed by the same experienced orthopedic surgeon using the standard TTA technique as described by Montavon in 2004 [7], with a plate and screw set in place of a fork set; cages of different widths (6 mm, 7.5 mm, 9 mm,10.5 and 12 mm) were used (Demas srl, Rome, Italy). Based on clinical and/or ultrasound findings of no concomitant meniscal lesions, arthrotomy, meniscectomy or release were not performed.

After surgery, a modified Robert Jones bandage was applied to the limb for 24 hours. Exercise was strongly restricted until suture removal and was then controlled pending the 2-month follow-up.

### Radiographic procedures

Computed radiographic images (FCR Capsula, Fujifilm, Italy) were used to carry out all the radiographic studies, and a specific software program (Onis 2.5 software, DigitalCore Co, Tokyo, Japan) was used to evaluate the DICOM images.

The mediolateral projections were obtained with the joint flexed at 135°, directing the X-ray beam to the center of the medial femoral epicondyle, with superimposition of the medial and lateral condyles; a tolerance of 2 mm of condyle non-overlap was fixed [21,27].

Evaluation of the signs and progression of OA in the stifle joints was carried out by two veterinarians (a PhD Diagnostic Imaging Postgraduate and an experienced radiologist) who were blinded regarding the different time points. The worst OA score assessed by each radiologist was selected for the statistical analysis.

**Fig 1:**
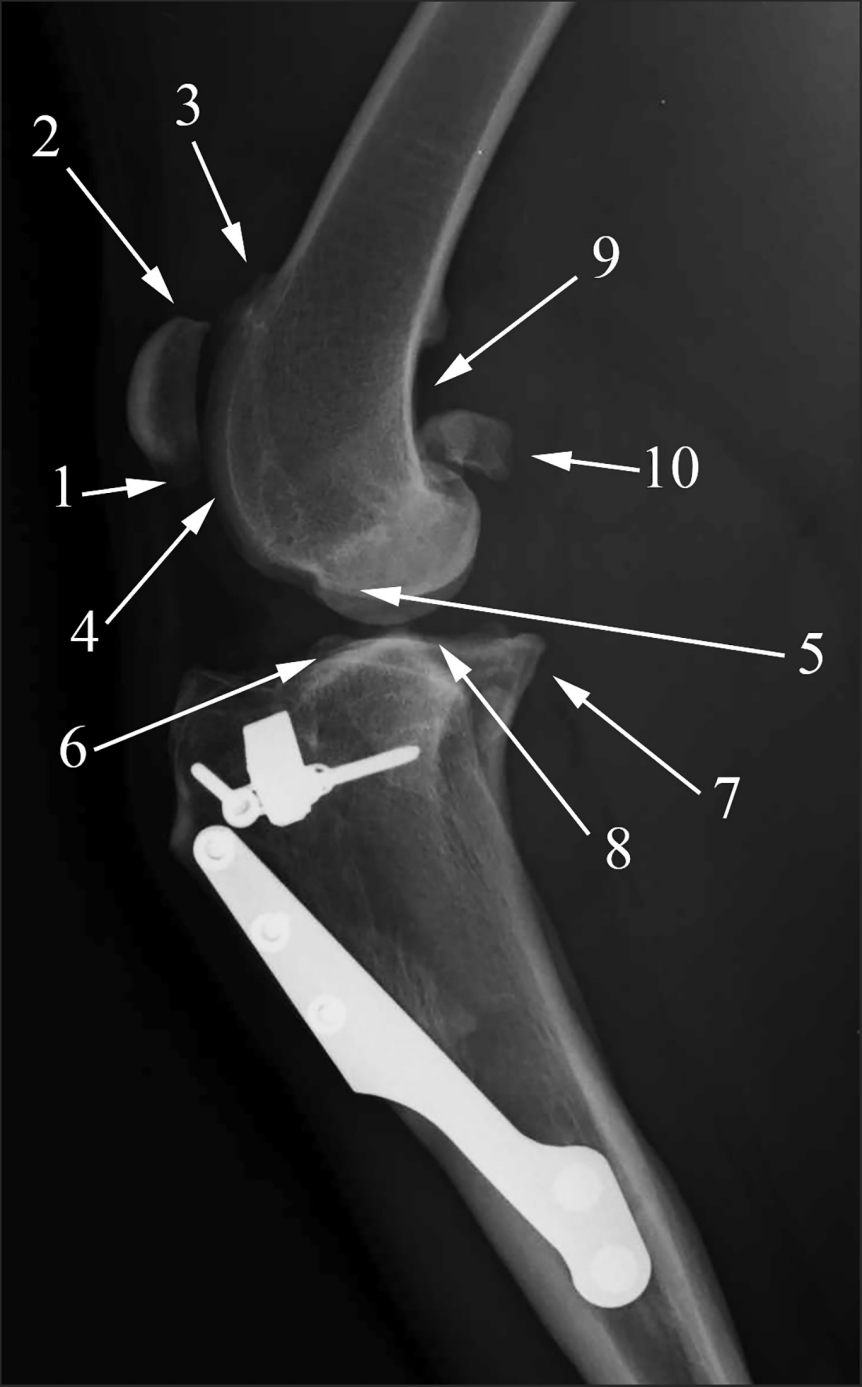
Anatomic locations for the assessment of osteoarthritis. Radiographic images of the right stifle joint of a 3-year-old 27 kg male Boxer at 6 months after a tibial tuberosity procedure. 1) patellar apex, 2) patellar base, 3) proximal trochlear ridge, 4) distal trochlear ridge, 5) femoral condyle, 6) cranial aspect of the tibial plateau, 7) caudal aspect of the tibial plateau, 8) central aspect of the tibial plateau, 9) popliteal surface of the femur and 10) sesamoid bones.

Assessment of the OA was achieved considering the presence of osteophytosis and subchondral sclerosis in10 specific anatomic locations of the stifle joint (Fig 1). The presence of an osteophyte is considered to be one of the defining features of OA in animals [28].

For each location, a score from 0 to 3 based on the amount of new bone and bone density (0 = no signs of OA; 1 = mild-OA: early osteophytes and/or sclerosis; 2 = moderate-OA: obvious osteophytes and subchondral sclerosis; 3 = severe-OA: marked osteophytes and severe subchondral sclerosis) was assigned [20]. The stifle OA scoring system was modified in this study and a score of 0 to 3 was used in place of the 1 to 4 in Wassely’s study [20]

The global OA score of each radiograph was calculated as the sum of the scores obtained for each location (global scores from 0 to 30) at each time point. The dogs were divided into 4 groups based on the scoring intervals: 0–3 (Group A: no-OA), 4–10 (Group B: mild-OA), 11–20 (Group C: moderate-OA) and 21–30 (Group D: severe-OA).

In addition, two groups were created on the basis of cage size: cages less than 9 mm wide (Small Cage group) and cages greater than 9 mm wide (Large Cage group).

Finally, each anatomic location was evaluated at each time point, and the data were used in order to find the site of the greatest OA changes.

### Statistical analysis

Breed, gender, age, weight and limb data were collected. The results from the descriptive statistics were reported as mean, standard deviation (SD) and range.

The data were analyzed using a statistical software program (MedCalc Software 16.8.4, Ostend, Belgium). Normal distribution was assessed by means of Levene’s Test (*P* > 0.05). An analysis of variance (ANOVA) for repeated measures was used to compare the scores of the OA during the five evaluations (the Bonferroni correction was used as a post-hoc analysis). The Kruskall-Wallis test was used to investigate the changes in the OA scores for non-parametric data (rejected normality), and the Chi-squared test was used to compare the distribution of the categorical data. The weighted kappa statistic (κ), with 95% confidence interval (CI) [29], was used to assess the agreement between the two radiologists for the global stifle score of OA.

The dogs were also analyzed individually to identify OA progression of at least one score interval, and to find a correlation between the OA changes and both the duration of lameness and the size (width) of the cage employed. Significance for all the analyses was set at *P* < 0.05.

## Results

Thirty-eight dogs (38 stifle joints) met the inclusion criteria for the study, and a total of 190 X-ray images were collected. The most common breeds included mixed-breed dogs (n = 9), American Staffordshire Terriers (n = 5), Labrador Retrievers (n = 6) and Cane Corsos (n = 3); other breeds were Australian Shepherds, Beagles, Bernese Mountain Dogs, Border Collies, Boxers, Dogues de Borgeaux, German Shepherd Dogs, Golden Retrievers, Maremma Sheepdogs and Rottweilers (n = 1 or 2 dogs/breed).

There were 13 intact males and 3 neutered males, 16 unspayed females and 6 spayed females. The mean age was 5.9 yrs ± 2.5 (range: 2–13 yrs) and the mean body weight was 34 kg ± 9.5 (range: 15–54 kg). The mean duration of lameness prior to surgery was 86.5 days ± 98.6 (range: 1–365 days), 20 dogs presented lameness of 15.3 days ± 11.8 (range: 1–30 days) duration (short term: < 30 days) and 18 dogs showed lameness of 165.5 days ± 91.7 (range: 60–365 days) duration (medium-long term: > 30 days).

The TTA procedure was performed on 16 right stifle joints and on 22 left stifle joints.

The widths (mm) of the cages used were: 6 mm (n = 8), 7.5 mm, (n = 5), 9 mm (n = 9), 10.5 mm (n = 12) and 12 mm (n = 4).

The mean of the OA global scores of the 38 stifle joints was 6 ± 0.5 (range: 0–17) at T0 and 8.9 ± 5.4 (range: 0–19) at T6. The inter-observer agreement for the OA score was good, with κ = 0.84 (95% CI 0.70–0.99).

The number of stifle joints in the groups according to the scoring intervals of OA were listed in Table 1. The OA scores were statistically significant (*P* < 0.001) between the groups. Significant differences were not found between the groups in the distribution of gender, age and body weight. There was a correlation between the duration of lameness and the severity of OA (*P* = 0.04).

**Table 1:** Distribution of stifles according to the duration of lameness assessed prior to surgery and statistical details of the OA scores in the groups.

Sample	Duration of lameness		OA score Mean ± SD (range)				
	< 30 days n. (%)	> 30 days n. (%)	T0	T1	T2	T3	T6
Group A (n = 13)	10 (26.3)	3 (7.9)	1.2 ± 1.4 (0–3)	1.4 ± 1.7 (0–4)	2 ± 2 (0–7)	2.7 ± 2.4 (0–7)	3.5 ± 2.8 (0–10)
Group B (n = 19)	9 (23.7)	10 (26.3)	6.6 ± 2.1 (4–10)	6.7 ± 2.1 (4–10)	7.7 ± 2 (5–11)	8.4 ± 2.7 (5–16)	10.3 ± 3.7 (5–16)*
Group C (n = 6)	1 (2.6)	5 (13.2)	14 ± 1.8 (12–17)	14 ± 1.8 (12–17)	14.7 ± 1.9 (12–17)	15.3 ± 1.9 (12–17)	16.3 ± 1.8 (14–19)
Group D (n = 0)	NA	NA	NA	NA	NA	NA	NA

T0: baseline prior to surgery; T1, T2, T3 and T6 months after surgery; NA: not applicable

* Statistically significant

**Fig 2:**
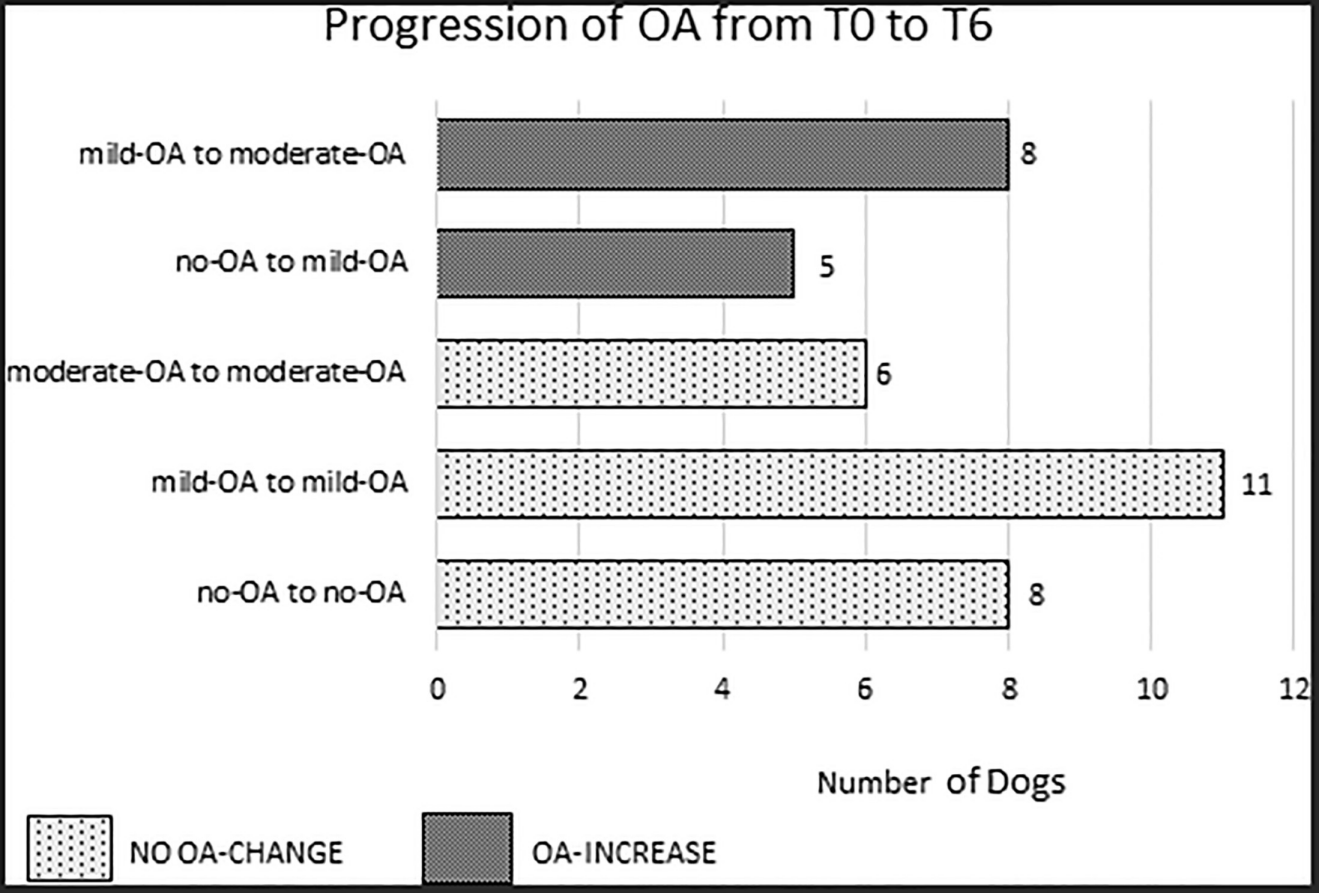
Progression of the osteoarthritis scores from the time of surgery (T0) to six months (T6).

The OA assessment based on the radiographs of all 38 stifle joints showed a significant increase in the global scores at T2, T3 and T6 as compared with the baseline values (*P* < 0.001). Nevertheless, an overall evaluation revealed that 25 (65.8%) of the 38 stifle joints preserved their stage of OA between the preoperative and postoperative time periods, and only 13 (34.2%) of the 38 stifles increased their stage of OA by 1 score interval (5 stifles from no-OA to mild-OA and 8 stifles from mild-OA to moderate-OA). No severe-OA was recorded (Fig 2).

Investigation of the OA changes in the aforementioned groups revealed that, in Group A and in Group C, the OA progression was not statistically significant at each pairwise comparison (*P* > 0.05); however, in Group B, there were significant OA changes from T0 to T3 and T6 (*P* < 0.001) (Table 1).

Twenty-two out of the 38 stifle joints were in the Small Cage group and 16 of the 38 stifles were in the Large Cage group. Analysis of contingency table did not find any correlation between the size of the cage used to advance the tibial tuberosity and the change in OA in either group (*P* > 0.05). Moreover, the OA scores were not significantly different between the small and the large cage groups at either time point (*P* = 0.64 at T0 and *P* = 0.88 at T6). No correlation was found between the size of the cages and the weight of the dogs: very weak (r_s_ 0.18; *P* = 0.43) and weak (r_s_ 0.29; *P* = 0.36) in small and large cage groups, respectively.

The analysis of the scores in each anatomic location revealed that the OA was already evident prior to surgery (T0) in the patella apex, in the proximal and distal trochlear ridge, and in the caudal aspect of the tibial plateau. The OA in these locations was greater than in the other locations (mean scores > 0.7). After the TTA procedure, the distal trochlear ridge, the femoral condyle and the caudal aspect of the tibial plateau revealed significant OA progression at T3. Furthermore the OA progression was statistically significant at T6 (*P* < 0.05) in all locations, except for the cranial aspect of the tibial plateau, the popliteal surface femur and the sesamoid bones in which the OA changes were not significant (Table 2).

**Table 2:** Progression of OA. Mean ± SD scores for each location assessed in order to calculate the radiographic OA scores in 38 dogs with CCL rupture prior to and after TTA surgical repair. Number and percentage of stifle joints which showed changes in OA from T0 to T6.

	Mean ± SD					*P* -value		Joints with changes n. (%)
Locations*	T0	T1	T2	T3	T6	T3	T6	from T0 to T6
1	1.1 ± 0.8	1.1 ± 0.8	1.1 ± 0.8	1.2 ± 0.8	1.3 ± 0.8§		0.0136	10 (26.3)
2	0.5 ± 0.6	0.6 ± 0.6	0.6 ± 0.6	0.7 ± 0.6	0.8 ± 0.7§		0.0136	10 (26.3)
3	1.1 ± 0.9	1.1 ± 0.9	1.3 ± 0.9	1.3 ± 0.9	1.4 ± 0.9§		0.0168	9 (23.7)
4	0.7 ± 0.9	0.7 ± 0.9	0.8 ± 0.9	0.9 ± 0.9†	1.0 ± 0.9§	0.033	0.0069	11 (28.9)
5	0.5 ± 0.6	0.5 ± 0.6	0.6 ± 0.8	0.7 ± 0.8†	0.8 ± 1.0§	0.033	0.0088	11 (28.9)
6	0.3 ± 0.6	0.3 ± 0.6	0.4 ± 0.6	0.4 ± 0.6	0.5 ± 0.6		NS	6 (15.8)
7	0.7 ± 0.7	0.7 ± 0.7	0.8 ± 0.8	1.0 ± 0.8	1.4 ± 0.9§	0.0009	<0.0001	23 (60.5)
8	0.2 ± 0.4	0.2 ± 0.4	0.3 ± 0.5	0.3 ± 0.5	0.5 ± 0.6§		0.0136	10 (26.3)
9	0.3 ± 0.5	0.3 ± 0.5	0.4 ± 0.5	0.4 ± 0.6	0.5 ± 0.6		NS	4 (10.5)
10	0.6 ± 0.6	0.6 ± 0.6	0.6 ± 0.6	0.6 ± 0.7†	0.8 ± 0.7		NS	8 (21.1)

OA: osteoarthritis; CCL: cranial cruciate ligament; TTA: tibial tuberosity advancement; SD: standard deviation; NS: not significant

*see Fig 1

^†^ Statistically significant at T3

^§^ Statistically significant at T6

## Discussion

The results obtained from this study confirmed the authors’ hypothesis: the preoperative OA score influenced the progression of the disease, and the TTA procedure carried out in dogs in the absence of or in the early stages of OA could slow the OA evolution.

In order to attempt comparison of the findings of the present study to those of other studies following TPLO/TTA, the validated OA stifle scoring system described by Wassely [20] was used. Reproducibility, feasibility and accuracy of the scoring methodology was assessed [20]. In the first step of the present study, the radiographic evaluation was carried out on the overall sample without distinction of the severity of OA prior to the surgery. It was found that 34.2% of the stifles treated had OA progression, which was less than the results of other studies performed following both TTA [17,18] and TPLO [30].

Subsequently, the groups were evaluated based on OA scores. As expected, the dogs without signs of OA (Group A) did not show statistically significant OA progression after TTA for treatment of CCL rupture. The literature has reported that joint stability could play an important role in the reduction of OA development, sustaining the goal of repairing the CCL rupture in order to restore function and prevent subsequent damage to the articular cartilage and the periarticular structures [31]. Inversely, it is reasonable to suppose that the limited OA progression obtained in the group without initial OA could be due to the effectiveness of the surgical technique, but additional studies are necessary to sustain this supposition.

In the present study, progression was small, but significant, in the stifle joints with mild OA prior to surgery (Group B). There was an increase in the severity of radiographic signs of OA in less than half (8 stifles out of 19) similar to other reports [17,18,30]. The evaluation of OA in the stifle joints with moderate OA (Group C) revealed that the OA changes were not statistically significant at each pairwise comparison over time. The results obtained in both Groups B and C agree with the inverse relationship between the preoperative OA score and the difference between pre and postoperative OA scores as reported in the literature [32,33]; however, a similar relationship it was not found in the stifles without OA in Group A. Furthermore, the group of moderate OA was composed of dogs with a longer duration of lameness. It was supposed that when a ligament lesion is chronic and the OA is advanced and has most likely reached a plateau prior to surgery, the progression of the cartilage degeneration of the joint is slow and the potential advancement of the OA is minimal [32].

The OA development is attributed to several variables. Age, body weight, duration of lameness, meniscal injury, and surgical procedure such as meniscal release, meniscectomy and arthrotomy, have been described as potential risk factors for the development of OA in an affected stifle joint [18,30,32,34–37].

Both meniscectomy and meniscal release had an effect on the progression of the osteoarthritis [36,37] so much so that, in the case of a subclinical meniscal tear, a conservative approach could also be valid [38]. Previous studies regarding the radiographic evaluation of OA in dogs treated with osteotomy procedures found high scores of OA before surgery in the stifles with meniscal injury, and those who had had a meniscectomy had a faster progression of OA [18,30].

An arthrotomy procedure could also influence OA changes. Open arthrotomy can result in high morbidity in clinical outcomes and predispose to increasing OA progression as compared to both arthroscopy and limited caudal arthrotomy [32,35]. It has been reported in the literature that an arthrotomy performed without experimental transection of the CCL led to OA progression due to the activation of inflammation from the surgical procedure [39].

In the present study some limitations should be noted. This retrospective study was designed in an attempt to contain the incidence of the aforementioned. The first limitation was that only dogs with intact menisci were enrolled in the study. Dogs which had undergone open arthrotomy, meniscectomy and meniscal release did not meet the inclusion criteria. The aim of this rigid process of the choice of dogs was to select a homogenous group to study and to assess the effects of TTA on OA development. It is well known that this would not reflect the real population of CCL rupture in dogs. The second limitation was that the diagnosis of intact menisci was reached on the basis of clinical and/or ultrasonography evaluations rather than on arthroscopy or open arthrotomy. Although clinical and ultrasonography examinations have good sensitivity and specificity (24,26) some meniscal injuries could be mis-diagnosed. However, in the present study, the absence of lameness or major surgical complications, such as a subsequent meniscal tear, during the 12-month follow-up, could suggest the accuracy of the meniscal evaluation. The third limitation of this study was that dogs with suspicion of a partial tear of the CCL were excluded. Aware of a possible mis-diagnosis, the cranial drawer shown in both flexion and extension as a sign of complete rupture of CCL was the first point for the recruitment of the dogs. Dogs with a cranial drawer presented only in flexion were excluded, being indicative of torn craniomedial band and an intact caudolateral part [40]. Finally, in the present study therapies with nutraceuticals and anti-inflammatory drugs, and/or rehabilitation therapy were not assessed, and additional studies to evaluate their effects on OA changes should be carried out.

As expected, a direct correlation between the severity of OA score and the duration of lameness was found in the present study. In the literature, there are some discrepancies in this correlation. Hoffmann reported that the length of lameness time contributed to the OA score on the preoperative radiographs [17], contrary to Bennett’s study which did not find an obvious correlation between the degree of osteoarthritic change and the duration of the lameness [13]. According to the literature, OA is a degenerative and progressive pathology and it is the cause of lameness in dogs [41], even though it has previously been reported that there is no relationship between the functional capacity of dogs with OA and radiographic evidence of disease [42]. In the present study, prevalence among those with short and long-term lameness was not observed in the dogs with mild OA; this could probably have been indicative of a transition stage between the absence of and moderate OA.

In the present study, no correlation was found between OA development and the size of the cage used to advance the tibial tuberosity. This finding could be suggestive of the accuracy of the preoperative planning and support the biomechanical concept of neutralization of the cranial tibial thrust force [4,9,43]. Despite this, a later report has shown that cranial tibial subluxation persisted in the majority of the stifle joints evaluated, but the majority of the dogs returned to good limb function regardless of the femorotibial alignment [44]. In that study, a medial meniscectomy had been performed in 90% of the dogs, but the menisci had been identified as important stabilizers of the stifle in ex vivo studies in dogs [44–46]. This statement was an additional reason for recruiting only dogs with intact menisci in the present study.

In order to obtain more information, the identification of the anatomic locations in the stifle joint in which the greatest progression of OA appeared after surgical repair of the CCL rupture with a TTA procedure was carried out. The authors of the present study agreed with the literature regarding the sequence of the appearance of osteophytes in the specific locations of the stifle joints with CCL rupture. In particular, at the beginning of this study (T0), the OA signs were already evident in the patellar apex, and the proximal and distal trochlear ridges in which the periarticular osteophytes appeared early in the stifles with CCL rupture [10,14,15,47]. In the caudal aspect of the tibial plateau, a high mean of the OA score was also found which could have been indicative of osteophytes growth as a reaction to joint instability [16]. This was in agreement with the findings of other authors obtained at 6–12 weeks after experimental transection of the CCL [15], and with a study regarding the supposed microinstability of the contralateral stifle joints [14].

The patellar apex, the proximal and distal trochlear ridges and the caudal aspect of the tibial plateau showed a significant increase in OA six months (T6) after surgery, but an individual evaluation revealed that the changes were produced in only a small number of cases. These results could be explained on the basis of the following observations.

First, the increasing tension on the patellar tendon consequent to the CCL rupture could have been the cause of the early OA signs in the patellar apex. There are no biomechanical studies which demonstrate the influence of the TTA on tendinous stress, but it has been hypothesized that the altered anatomy created by the TTA procedure could lengthen the lever arm and decrease the stress on the patellar tendon [9,48,49]. This decreased stress could reduce the tension of the tendon in the patellar apex, in fact, in the present study, the OA did not worsen in 28 (73.7%) of the 38 stifle joints in the patellar apex during the follow-up. This is a subjective supposition, therefore, additional studies are necessary to prove this biomechanical concept.

Second, similar results were found in the proximal and distal trochlear ridges in which 29 (76.3%) and 27 (71.1%) of the 38 stifles, respectively, preserved the score from T0 to T6. As a result of the TTA procedure, the patella is distanced from the trochlear groove; thus the retropatellar force on the cartilage of the trochlear groove decreased [49–51]. The authors assumed that the reduction of the pressure would have decelerated OA progression in both the proximal and the distal trochlear ridges.

Third, the different trend in the caudal aspect of the tibial plateau in which the OA increased in 23 (60.5%) of the 38 stifles was interesting. Although the modification of the stifle geometry obtained with the TTA procedure has the aim of neutralizing the cranial tibial subluxation [9], it does not restore the position of the tibia in relation to the femur. Therefore, in the present study, it was hypothesized that this misalignment could be the reason for the progression of the OA in the caudal aspect of the tibial plateau in agreement with Leach’s study [52] in which the distribution of the femorotibial contact after TPLO and TTA was investigated. Additional biomechanical studies are necessary to confirm these statements.

In conclusion, the results of this study suggested that the TTA procedure could reduce the development of OA for least 6 months after surgery when performed on dogs without either meniscal lesions and radiographic signs of OA or with OA in an early stage at the time of surgery. In agreement with the literature, the TTA procedure carried out in dogs with mild OA did not halt OA progression but it advanced slowly over time without reaching high scores. The ability of the TTA procedure to reduce the progression of OA is obvious in cases of moderate OA at the time of the surgical treatment, in which no changes in the stage of OA were recorded after surgery.

In light of these findings, it is reasonable to assume that the initial stage of osteoarthritis can influence the effectiveness of the TTA procedure; however, additional studies should be carried out to evaluate the effects of TTA over the long-term.
